# Does Rubella Immunity Predict Measles Immunity? A Serosurvey of Pregnant Women

**DOI:** 10.1155/IDOG/2006/13890

**Published:** 2006-07-13

**Authors:** Colleen M. Kennedy, Barbara A. Burns, Kevin A. Ault

**Affiliations:** ^1^Department of Obstetrics and Gynecology, Roy J and Lucille A Carver College of Medicine, University of Iowa Hospitals and Clinics, Iowa City, IA 52242, USA; ^2^Department of Gynecology and Obstetrics, Emory University School of Medicine, Atlanta, GA 30322, USA

## Abstract

*Background*. This study was undertaken to determine whether rubella immunity infers measles immunity in pregnant women. *Methods*. Stored serum samples were obtained from the Iowa State Hygienic Laboratory for evaluation of rubella and measles immunities with IgG enzyme-linked immunosorbent assay. *Results*. Nine hundred serum samples were obtained for testing. The average age of the women at the time of antepartum serum collection was 28 (range, 14 to 44) years. Measles and rubella immunity were 88% and 98%, respectively; there was no effect of immunity status by age identified. Eighty eight percent of those with rubella immunity were also measles immune. There was no association between paired rubella and measles immunity identified, *P* < .0001. *Discussion*. Known rubella immunity did not infer measles immunity in our population. Thus, we recommend that pregnant women exposed to measles be tested and appropriately treated if they are found to be nonimmune.

## INTRODUCTION

Given a renewed public awareness following a recent measles outbreak in Iowa, several pregnant women inquired about their measles immunity [1]. While confirmation of measles immunity is not routinely performed, determination of rubella immunity is a routine antenatal test in the United States. Consequently, we questioned whether a woman with known rubella immunity would be likely to have measles immunity as well.

A preliminary review of the literature confirmed that measles immunity following vaccination is reported to be from 95% (1 dose) to 99% (2 doses), and rubella immunity is reported to be 85% to 90% among adults [2]. While both vaccines have a high immunogenicity, rubella immunity is shown to be somewhat lower than measles immunity. Thus, depending upon the paired association for immunity, rubella immunity could be useful as a predictor for measles immunity among women with known rubella immune status. While correlation of rubella and measles immunity has been reported, the more rigorous statistical tests to determine paired association have not.

## METHODS

Serum was obtained from the Iowa State Hygienic Laboratory from samples collected between January of 2004 and November of 2004 among women seeking antenatal care in Iowa and submitted for routine testing (hepatitis B). The University of Iowa Internal Review Board approved the study.

The sample size for the study was estimated based on the primary outcome measure, agreement between measles and rubella immunity in a population serum sample. The sample size was calculated using McNemar's test (paired data) with level of significance 0.05, power 80%, rubella immunity 85%, and measles immunity 97%, which determined that 867 samples would be required to determine a paired association of immunity status.

Rubella immunity was determined by a commercially available rubella IgG enzyme-linked immunosorbent assay (ELISA) (BIO-QUANT, Inc., NY, USA). Likewise, measles (rubeola) immunity was determined with a commercially available measles IgG ELISA (BIO-QUANT, Inc.).

Statistical analyses were conducted using the Statistical Analysis System version 9.0 (SAS, NC, USA) to describe the rates of measles and rubella immunity. The Kappa statistic was utilized to assess concordance of immunity status. The paired data for each serum sample was evaluated using McNemar's test to evaluate whether or not there was a paired association between immunity statuses. The Wilcoxon rank-sum test was used for nonparametric comparison of the age means between immune and nonimmune individuals.

## RESULTS

Nine hundred serum samples were obtained and tested for both measles and rubella immunities. The age of the women was known for 785 samples, with the average age 28 (range 14 to 44) years. Demographic data beyond age was unknown. However, the population of Iowa is primarily non-Hispanic white.

As noted in Table 1, of the 900 samples tested, 790 were immune, 69 were nonimmune, and 41 were indeterminate to measles. Similar testing found that 883 samples were immune, and 17 were nonimmune to rubella (none of the rubella tests were indeterminate). All of the measles indeterminate samples were found to be rubella immune. Immunity to measles and rubella was found to be 88% and 98%, respectively.

Measles immunity status was noted for each rubella immune and rubella nonimmune sample groups to determine the association of immunity status. The probability of measles immunity given that a sample was found to be rubella immune was 88%. There was no concordance between immunity statuses, Kappa 0.1353 (95% CI 0.0314, 0.2392). Additionally, McNemar's test rejected a paired association between measles and rubella immunities, *P* < .0001. Even if all the serum samples found to be indeterminate for measles immunity were found to be measles immune, neither concordance nor paired association would have been confirmed (Kappa 0.1366, McNemar's *P* < .0001). The mean age of those women in each rubella group (immune and nonimmune) was the same (28 years). Thus, there was no apparent effect of immunity status by age identified.

The measles and rubella immunities prevalence identified in the population studied was different than reported in the literature (noted previously). Therefore, a posthoc analysis was performed to determine power for the identified measles and rubella immunities prevalence in our population using McNemar's exact conditional test, with a computed power of > 0.999, and 0.0458 level of significance.

## DISCUSSION

In 2005, the CDC independent panel concluded unanimously that rubella was no longer endemic in the United States [3]. Unfortunately, there is still a significant minority of reproductive age women who are rubella susceptible. The goal of prenatal testing is to identify women for vaccination in the postpartum period as the measles-mumps-rubella (MMR) vaccine is contraindicated in pregnancy.

The rubella vaccine was licensed in 1969. Since 1969, rubella-associated morbidity and mortality and the incidence of congenital rubella syndrome have greatly declined [2]. The rubella vaccine has been administered as part of the MMR vaccination since 1978. In 1990, a two-dose schedule was adopted (age 15 months and again at age 4–6 years). Following vaccination, measurable antibodies are present in 95% of individuals. Lasting immunity is present in 82% to 90% of those who initially seroconverted using the two-dose regime [4].

The measles vaccine was licensed in 1963. Since 1963, there has been a 99% reduction in the incidence of measles in the United States [2]. Unlike rubella, antepartum measles infection has no consistent pattern of fetal anomalies. However, there is a known increase in spontaneous abortions, premature births, and maternal morbidity, including pneumonia and encephalitis. Passive immunization within six days of exposure is recommended in pregnant women [2].

We found that rubella immunity did not infer measles immunity in our study population. While correlation has been reported by others [5, 6] and was also noted in this study, correlation does not imply the more rigorous statistical associations of agreement or concordance. The large number of serum samples positive for both rubella and measles resulted in the correlation we identified, as would be expected in an immunized population.

Strengths of our study include the large sample size, and prospective data analysis. A limitation of our study was that the serum samples were obtained from Midwest (primarily Caucasian) pregnant women, which limit generalizability. However, our findings agree with large military studies where participants included both men and women from across the United States with varying ethnic background and race [4, 7, 8].

Measles immunity was found to be 88% and rubella immunity 98%. The immunity rates for measles and rubella may differ within the population we studied compared to those previously reported. Alternatively, the assay for measles antibody could be less sensitive than the assay for rubella antibody. This would be consistent with the high number of measles indeterminate results noted and could be related to the greater number of nonimmune measles results. Further Investigation may be undertaken to address this possibility.

In conclusion, rubella immunity did not infer measles immunity in our population. In measles outbreaks as that in 2004, we would be unable to presume a women's measles immunity based on known rubella immunity. Thus, pregnant women exposed to measles should be tested and treated if nonimmune.

## Figures and Tables

**Table 1:** Measles and rubella immunities status.

	Measles		

Rubella	Immune	Not immune	Indeterminate
Immune	780	62	41
Not immune	10	7	0
